# Sociodemographic characteristics of missing data in digital phenotyping

**DOI:** 10.1038/s41598-021-94516-7

**Published:** 2021-07-29

**Authors:** Mathew V. Kiang, Jarvis T. Chen, Nancy Krieger, Caroline O. Buckee, Monica J. Alexander, Justin T. Baker, Randy L. Buckner, Garth Coombs, Janet W. Rich-Edwards, Kenzie W. Carlson, Jukka-Pekka Onnela

**Affiliations:** 1 Department of Epidemiology and Population Health, Stanford University School of Medicine, Stanford, CA USA; 2 Department of Social and Behavioral Sciences, Harvard T.H. Chan School of Public Health, Boston, MA USA; 3 Department of Epidemiology, Harvard T.H. Chan School of Public Health, Boston, MA USA; 4 Department of Sociology, University of Toronto, Toronto, ON Canada; 5 Department of Statistical Sciences, University of Toronto, Toronto, ON Canada; 6 Department of Psychiatry, Harvard Medical School, Boston, MA USA; 7 Institute for Technology in Psychiatry, McLean Hospital, Belmont, MA USA; 8 Department of Psychology, Harvard University, Cambridge, MA USA; 9 Department of Psychiatry, Massachusetts General Hospital, Boston, MA USA; 10 Department of Radiology, Massachusetts General Hospital, Boston, MA USA; 11 Division of Women’s Health, Department of Medicine, Brigham and Women’s Hospital and Harvard Medical, Boston, MA USA; 12 Department of Biostatistics, Harvard T.H. Chan School of Public Health, Boston, MA USA

**Keywords:** Epidemiology, Public health

## Abstract

The ubiquity of smartphones, with their increasingly sophisticated array of sensors, presents an unprecedented opportunity for researchers to collect longitudinal, diverse, temporally-dense data about human behavior while minimizing participant burden. Researchers increasingly make use of smartphones for “digital phenotyping,” the collection and analysis of raw phone sensor and log data to study the lived experiences of subjects in their natural environments using their own devices. While digital phenotyping has shown promise in fields such as psychiatry and neuroscience, there are fundamental gaps in our knowledge about data collection and non-collection (i.e., missing data) in smartphone-based digital phenotyping. In this meta-study using individual-level data from six different studies, we examined accelerometer and GPS sensor data of 211 participants, amounting to 29,500 person-days of observation, using Bayesian hierarchical negative binomial regression with study- and user-level random intercepts. Sensitivity analyses including alternative model specification and stratified models were conducted. We found that iOS users had lower GPS non-collection than Android users. For GPS data, rates of non-collection did not differ by race/ethnicity, education, age, or gender. For accelerometer data, Black participants had higher rates of non-collection, but rates did not differ by sex, education, or age. For both sensors, non-collection increased by 0.5% to 0.9% per week. These results demonstrate the feasibility of using smartphone-based digital phenotyping across diverse populations, for extended periods of time, and within diverse cohorts. As smartphones become increasingly embedded in everyday life, the insights of this study will help guide the design, planning, and analysis of digital phenotyping studies.

## Introduction

The ubiquity of personal digital devices has resulted in a unique opportunity to collect and analyze unprecedented amounts of data, providing researchers with a promise of a more nuanced understanding of human behavior than ever before. This trend continues to accelerate as internet-connected personal devices become more prevalent, accessible, and embedded in everyday life^1^. According to a recent study, over half of the world population has internet access^2^. Over six billion smartphones are estimated to be in circulation^3^, making smartphones the fastest growing technology in history^4^. In the United States, smartphone ownership is currently estimated at 85%, up from just 35% in 2011^5^.

Leveraging the resulting data deluge to understand human behavior in a more granular and precise manner, public health researchers have created the field of “digital epidemiology”^6,7^. Defined as health-related research using data generated outside of the health system and for non-health-related research purposes, digital epidemiology has advanced our understanding of how health and collective human behavior interact. For example, mobile phone data from telecommunications providers have been used to quantify the impact of human mobility on malaria transmission^8^, seasonal dengue^9^, and access to health care^10^. Digital traces from smartphone applications have been used to track mobility during the COVID-19 pandemic^11^. Similarly, social media data have been used to predict Zika incidence^12^ and city-level influenza emergency department visits^13^.

While digital epidemiology focuses on patterns of collective human behavior, the concept of a “digital phenotype” to understand individual human behavior was introduced in 2015^14^. We have previously defined the creation of a digital phenotype, or digital phenotyp*ing*, as “the moment-by-moment quantification of the individual-level human phenotype in situ using data from personal digital devices,” in particular smartphones^15–17^. As with any scientific inquiry, measurement is vital, and these personal digital devices provide an unprecedented opportunity for precise measurement of human behavior, at fine spatiotemporal resolution, using existing consumer grade devices across large, diverse samples. This pairing of individual-level data collection and analysis creates a nuanced view of the participant’s daily^14^ lived experience. The goal of digital phenotyping is to provide more precise social, behavioral, and cognitive phenotypes for developing a better understanding of various diseases, potentially leading to the establishment of new disease subtypes in fields such as psychiatry and neurology. These more precise phenotypes could enable early and accurate detection of diseases, thus advancing the goals of precision medicine, and monitor treatment response in an unobtrusive manner while facilitating measurement-based care at scale^16^.

While still nascent, digital phenotyping has shown significant promise, especially in the field of mental health^18,19^. For example, several studies have found a link between individual-level mobility, estimated from smartphone GPS sensor data, and depressive symptoms^20^. Among schizophrenia patients, digital phenotyping has been shown to be acceptable to patients and potentially feasible for use in clinical practice^21^, predictive of schizophrenic relapse in a small pilot study^22^, and capable of providing scalable and affordable sleep monitoring^23^. Additionally, digital phenotyping has begun to branch out to other areas of population health research: understanding the daily behaviors of healthy undergraduate students^24^, evaluating the risk of disordered eating among women with and without histories of childhood trauma and food insecurity, monitoring patient recovery after cancer surgery^25^, and providing enhanced medical care within a cohort of patients with advanced cancer^26^. However, researchers have also called for a better understanding of how these data are collected^27^, greater emphasis on methodology and techniques for analyses of these data rather than just on the collection itself^16^, and, as with any new area of research, establishing more ethical standards and guidelines for data collection^28^.

While many platforms exist for collecting data from smartphones, we focus on studies using Beiwe, an open source research platform for smartphone-based digital phenotyping. The development of Beiwe started in 2013, and the first version of the platform was introduced in 2016 and is described in detail elsewhere^15^. Briefly, Beiwe is a scalable, globally deployable, cloud-based data collection and data analysis platform designed for smartphone-based digital phenotyping in biomedical settings. Some of its distinguishing features are the ability to collect raw sensor data rather than pre-packaged data summaries, support for Android and iOS devices, emphasis on reproducibility of research through sharing of study configuration files, and full back-end integration with the Forest data analysis library that consists of statistical and machine learning methods specifically developed for analyzing smartphone data. Beiwe has been released under the 3-clause BSD open source license, which enables researchers to modify and expand the capabilities of the platform to meet their own scientific needs (Supplementary Information Text S1). Among other features, the platform allows investigators to specify which data streams are collected, how frequently they are sampled, and how frequently the data are uploaded to the server. Data are encrypted while buffered on the phone awaiting upload, during transit, and while at rest on the server. The support for both iOS and Android devices covers an estimated 99% of the U.S. smartphone market^29^.

Despite the potential for scalable, affordable, intensive data collection with a beneficial impact on medicine and public health, many fundamental questions about digital phenotyping data collection remain unanswered at this early stage of the field. For example, previous research has noted differences in smartphone mean duration of usage by gender and primary purpose of phone usage by age^30^. While the demographic differences in phone usage are clear, albeit under-researched, it remains unclear how these demographic differences may affect levels of missingness in smartphone-based digital phenotyping data collection. This is an important unresolved question in the field because missingness in digital phenotyping data can undermine the usefulness of many medical or public health applications. Design-based mitigation of missing data is preferable to traditional statistical approaches that largely ignore the problem (e.g., setting data “quality” thresholds and discarding blocks of time with high missingness) or rely on strong assumptions about types of missingness and recording relevant observable factors (e.g., statistical modeling). With few exceptions, statistically principled imputation of digital phenotyping data does not yet exists^31,32^.

Missing data in digital phenotyping can divided into two categories: (1) *missingness by design* and (2) *missingness due to sensor non-collection*. Missingness by design is an intended result of the sensor sampling schedule as configured by the investigator. For example, to preserve phone battery, at the design stage an investigator might configure the GPS sensor to collect data for 1 min every 10 min. In contrast, missingness due to sensor non-collection results from technological and behavioral factors. For example, a participant may forget to charge their phone, disable the GPS, or uninstall the study application. The phone’s operating system may also limit sensor access during specific scenarios due to performance considerations. Because the technological factors causing sensor non-collection are usually proprietary and therefore unknown to the investigator, identifying sensor non-collection and characterizing its extent is crucial so that the investigator, at a minimum, can quantify the resulting additional uncertainty in downstream data analyses, and can also consider imputing missing data. For smartphone applications that alternate sensor sampling between an on-cycle (sensor collects data) and off-cycle (sensor does not collect data), the expected data volume is known at the design stage, which enables one to easily diagnose sensor non-collection. In the above example, collecting data from the GPS sensor every 10 min for 1 min at a time leads to a regular 10% sampling coverage of any time period, resulting in 2.4 h of data for every 24-h period, for example. While outside the scope of this paper, we note that missingness due to sensor non-collection can be further divided into subtypes, such as missing completely at random, missing at random, and not missing at random, and distinguishing between these missing data mechanisms is important at the data analysis stage^33^.

Using individual-level data from six independent studies, this meta-study focuses on sensor non-collection and seeks to address four fundamental questions about this type of missingness in digital phenotyping data collection from accelerometer and GPS sensors: (1) What is the expected rate of sensor non-collection for accelerometer and GPS in digital phenotyping studies? (2) To what extent does the rate of sensor non-collection vary over the study period? (3) How are rates of sensor non-collection correlated with phone type (specifically, operating system, i.e., Android vs. iOS) or common demographic characteristics of participants, such as gender, education, or age? (4) How much does sensor non-collection vary across individuals? As far as we know, this is the first systematic investigation of these issues in a cross-diagnostic cohort in digital phenotyping.

## Results

**Figure 1:**
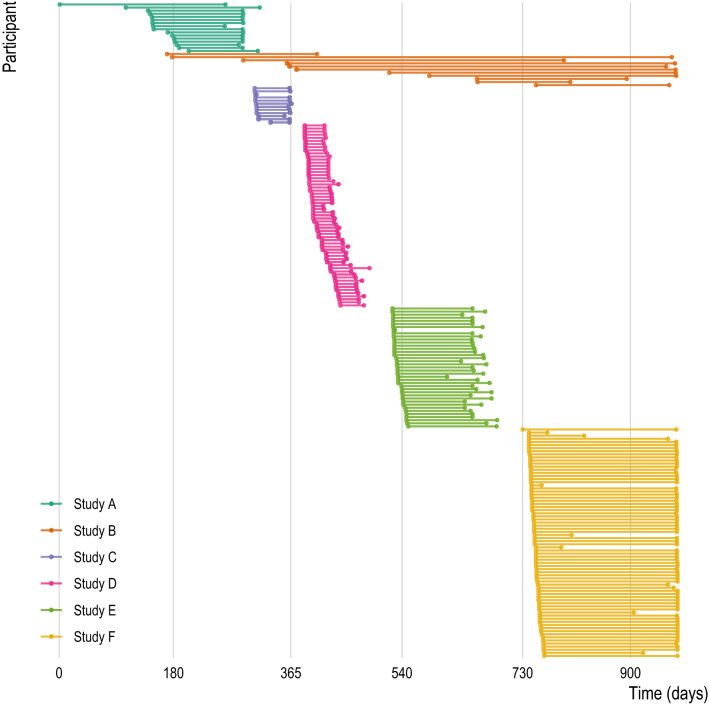
Periods of data collection for each study and each participant. Each horizontal line represents a single study participant with the endpoints at the first and last day of observation. Studies varied in number of participants, length of observation, and rate of attrition. Each study is represented by a different color. Note that because dates of study participation may be considered personally identifiable information, time (x-axis) is represented as the number of days relative to the first observation date in our data. All studies occurred between 2015 and 2018.

**Figure 2:**
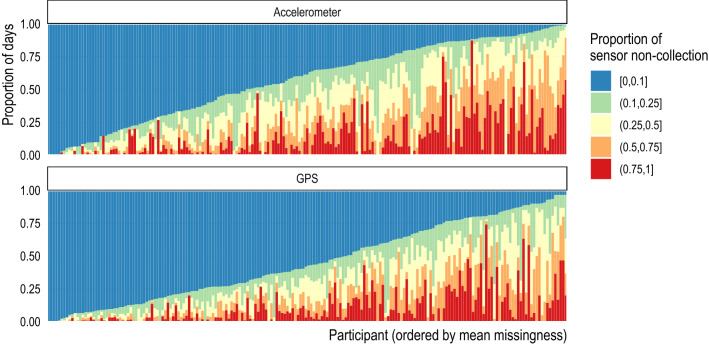
Proportion of missing observations by participant. Each vertical bar represents the proportion of missingness (color) for a single participant for accelerometer (top) and GPS (bottom) data. Participants are ordered by the average proportion of missingness. Follow-up was pre-specified in each study protocol based on time (i.e., not by the amount of data collected per subject).

In this meta-study, we analyzed the timestamps of accelerometer and GPS measurements collected in six different studies, conducted in 2015–2018, with a combined total of 211 participants (Figs. 1, 2, and S1) using the Beiwe Research Platform (Table 1). Measurements from accelerometer and GPS sensors occurred in the same individuals on the same phones, but were recorded independently (e.g., if GPS was disabled, accelerometer data would continue to be collected). In all, there were over 8.3 billion measurements (8.1 billion individual accelerometer measurements and 113 million GPS individual measurements) collected in over 81 million measurement groupings over the course of more than 29,500 person-days of observation (Table S1). For all analyses reported in this paper, we used only timestamps of each measurement (i.e., metadata) and not the measurement itself. Identifying information, such as GPS coordinates, were not necessary for the objectives of this study and thus all sensor measurements were removed before analysis. In addition to timestamps, we collected self-reported demographic information about participants in most of these studies (Table 1). These self-reported demographic data included gender, age, educational attainment (highest completed degree), and race/ethnicity (non-Hispanic White, non-Hispanic Black, Asian, American Indian/Alaska Native, other/Hispanic). Overall, among the 211 participants, the average age at the beginning of each study was 25.4 years (SD 10.8), most were female (66%), most had at most a high school education (67%), and 55% were non-Hispanic White, with the next two most common racial/ethnic groups being Asian (17%) and Black (14%). We note that because this is a meta-study, some descriptive statistics may have previously been published for individual studies, although none of these studies have specifically investigated missing data.

**Table 1:** Study demographic characteristics.

	Study A	Study B	Study C	Study D	Study E	Study F	Total (%)
Participants, N	16	11	12	59	39	74	211 (100%)
Mean (SD) age, y	19.4 (1.2)	31.5 (9.5)	20.4 (1.5)	41.1 (6.3)	18.4 (0.6)	18.2 (0.7)	25.4 (10.8)
**Phone OS, N**							
Android	0	7	12	40	35	69	163 (77%)
iOS	16	4	0	19	4	5	48 (23%)
**Gender, N**							
Male	4	8	5	0	16	36	69 (33%)
Female	12	3	7	57	23	38	140 (66%)
Missing	0	0	0	2	0	0	2 (1%)
**Education, N**							
High school	16	1	12	0	39	74	142 (67%)
Associates	0	6	0	3	0	0	9 (4%)
Bachelors	0	3	0	36	0	0	39 (18%)
Graduate degree	0	1	0	13	0	0	14 (7%)
Missing	0	0	0	7	0	0	7 (3%)
**Race/ethnicity, N**							
Non-Hispanic White	7	9	9	32	14	46	117 (55%)
Non-Hispanic Black	4	1	2	12	3	9	31 (15%)
Asian	5	1	1	7	14	9	37 (18%)
American Indian	0	0	0	0	0	2	2 (1%)
Other/Hispanic	0	0	0	5	5	5	15 (7%)
Missing	0	0	0	3	3	3	9 (4%)

General sociodemographic characteristics of each study and across all studies. Studies A, C, E, and F consisted of healthy undergraduate students from Harvard College. Study B consisted of patients known to be at risk for mania and psychosis from McLean Hospital in Massachusetts. Study D consisted of healthy female nurses from the Nurses’ Health Study 3. In parentheses, the Total column shows the row percent relative to the entire sample except for the age row where it shows the sample standard deviation of age in years.

**Figure 3:**
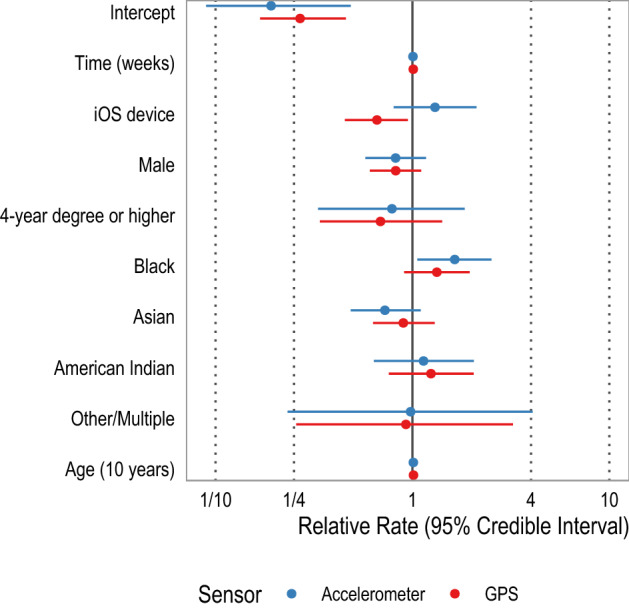
A forest plot of fixed effect estimates. The fixed effect estimates for accelerometer are in red and GPS in blue. Estimates have been exponentiated and can be interpreted as the relative change in sensor non-collection. The reference group for education is less than 4-year college degree and for race/ethnicity is non-Hispanic White. In terms of demographic characteristics, Black participants had higher rates of accelerometer non-collection compared to White participants; Asian participants had lower rates of accelerometer non-collection compared to White participants. iOS users had lower rates of GPS non-collection but higher rates of accelerometer non-collection, suggesting systematic differences in the phone operating systems of each phone.

We investigated the role of various sociodemographic characteristics for rates of sensor non-collection using Bayesian hierarchical negative binomial models detailed in "Methods". These models account for the correlated and nested nature of the data (i.e., observations within participants) and, unlike Poisson regression, allow for overdispersion of the data. The conditional average rates of sensor non-collection at the beginning of the studies were 19.1% (95% credible interval [CI]: 8.9, 45.8) for accelerometer and 26.9% (95% CI: 16.8, 45.9) for GPS (Table 2). The rates of sensor non-collection increased over time at approximately 0.5% (95% CI: 0.4, 0.7) per week for accelerometer and 0.9% (95% CI: 0.7, 1.0) per week for GPS (Table 2). Participants with iOS devices had substantially lower rates of GPS non-collection (RR: 0.66 [95% CI: 0.45, 0.95]) compared to participants with Android devices (Fig. 3).

**Table 2:** Model results.

*Fixed Effects*	Accelerometer		GPS	
	${\mathrm{e}}^{\upbeta }$ (95% CI)	SD	${\mathrm{e}}^{\upbeta }$ (95% CI)	SD
Intercept	0.191 (0.089, 0.485)	0.435	0.269 (0.168, 0.459)	0.255
Time (weeks)	**1.005 (1.004, 1.007)**	0.001	**1.009 (1.007, 1.010)**	0.001
iOS user	1.301 (0.803, 2.114)	0.246	**0.660 (0.453, 0.948)**	0.188
Male	0.821 (0.576, 1.171)	0.179	0.822 (0.607, 1.106)	0.152
4-year degree or higher	0.786 (0.332, 1.839)	0.437	0.688 (0.339, 1.416)	0.364
Non-Hispanic Black	**1.638 (1.059, 2.517)**	0.223	1.329 (0.907, 1.953)	0.196
Asian	0.724 (0.486, 1.100)	0.205	0.898 (0.630, 1.295)	0.183
American Indian	1.137 (0.637, 2.047)	0.298	1.241 (0.758, 2.044)	0.255
Other/Multiple	0.978 (0.232, 4.074)	0.727	0.926 (0.257, 3.240)	0.649
Age (10 years)	1.010 (0.973, 1.048)	0.019	1.011 (0.982, 1.042)	0.015

*Random Effects*	*SD* (*95% CI)*	Groups	*SD* (*95% CI)*	Groups
Level-1: Participant (${\sigma }_{\gamma }$)	1.012 (0.911, 1.127)	197	0.888 (0.799, 0.983)	197
Level-2: Study (${\sigma }_{\delta }$)	0.721 (0.222, 1.729)	6	0.295 (0.014, 0.906)	6

*Model*				
Observations (N)	28,218		28,053	
Shape $\omega$ (95% CI)	0.53 (0.52, 0.54)		0.64 (0.63, 0.65)	
Bayes R^2^ (95% CI)	0.384 (0.365, 0.403)		0.415 (0.391, 0.439)	
WAIC (SE)	430,853.5 (710.8)		216,522.5 (549.5)	
LOO (SE)	430,856.1 (710.9)		216,524.8 (549.6)	

Model estimates for all parameters for sensor non-collection rates of accelerometer (left) and GPS (right). The coefficients and 95% credible intervals (95% CI) have been exponentiated to assist interpretation. Parameters with 95% CIs that exclude 1 are in bold. The reference group for education is less than 4-year degree and that for race/ethnicity is non-Hispanic White.

In terms of accelerometer non-collection and demographic characteristics, there was no significant difference between male and female participants (RR: 0.82 [95% CI: 0.58, 1.17]) or participants with a four-year college degree compared to those without (RR: 0.724 [95% CI: 0.33, 1.84]). Similarly, rates of accelerometer non-collection did not increase with age (RR: 1.01 [95% CI: 0.97, 1.05]). Compared to White participants, Black participants had approximately 64% (95% CI: 5.9, 252) higher rates of accelerometer non-collection, albeit with substantial uncertainty. There was no similar difference for Asian participants (RR: 0.72 [95% CI: 0.49, 1.10]), American Indian or Alaska Native participants (RR: 1.14 [95% CI: 0.64, 2.05]), or participants of other racial/ethnic descent (RR: 0.98 [95% CI: 0.23, 4.07]). Unlike accelerometer, there were no statistically significant racial/ethnic differences in rates of GPS non-collection. There were no differences across any of the demographic characteristics for GPS non-collection: gender, race/ethnicity, education, or age (Table 2 and Fig. 3).

**Figure 4:**
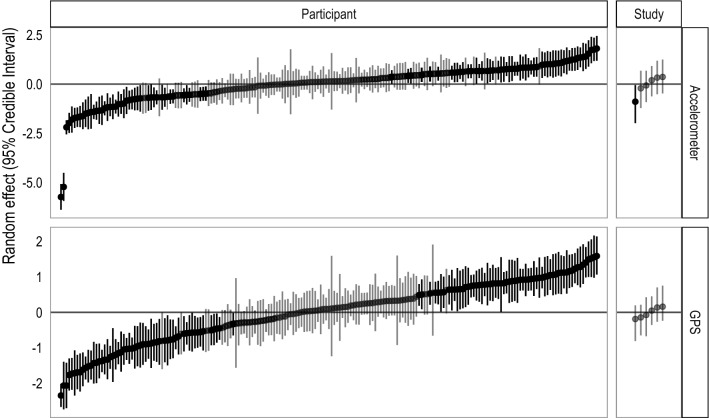
Participant-level (left) and study-level (right) random effect estimates for accelerometer (top) and GPS (bottom). The dots are the mean random effect estimates and the bars are the 95% credible intervals for each participant or study. Credible intervals that include 0 are shaded in grey while those that exclude 0 are shaded in black. The values on the y-axis represent the deviation from the overall average rate of sensor non-collection. There is substantial participant-level variation in missingness, and fairly low study-level variation relative to the participant-level variation. In all panels, estimates have been ordered from lowest (i.e., least sensor non-collection) to highest (most sensor non-collection) median value. Note that the y-axes differ across the rows.

Compared to other model specifications, the selected models provide the best goodness-of-fit while remaining parsimonious (Supplementary Information Text S2). Using Bayes R^2^, the proposed models explain 38% (95% CI: 37, 40) of the variance in the rate of accelerometer non-collection and 42% (95% CI: 39, 44) of the variance in the rate of GPS non-collection (Table 2). Additionally, individual-level variation was higher than study-level variation for both accelerometer (${\sigma }_{\gamma }$: 1.012 [95% CI: 0.95, 1.17] vs ${\sigma }_{\delta }$: 0.721 [95% CI: 0.222, 1.729]) and GPS (${\sigma }_{\gamma }$: 0.888 [95% CI: 0.81, 0.998] vs ${\sigma }_{\delta }$: 0.295 [95% CI: 0.014, 0.906])) non-collection (Fig. 4).

## Discussion

Our results suggest that overall sensor non-collection rates are 19% for accelerometer and 27% for GPS, with lower GPS non-collection among iOS users. In general, sensor non-collection did not vary by gender, age, or education. Accelerometer non-collection among Black participants is slightly higher relative to White participants, and no racial/ethnic differences were observed for GPS non-collection. Importantly, while there is a statistically significant temporal trend of increasing sensor non-collection, the size of the effect is small (~ 0.5–0.9% per week) and unlikely to be consequential in most studies relative to the baseline level of sensor non-collection. Lastly, we find larger variation in the amount of sensor non-collection at the participant-level relative to the study-level.

Our results have important implications for the design and analysis of future digital phenotyping studies. First, we show there is a nontrivial level of sensor non-collection across a variety of study settings and demographic characteristics. Future research in digital phenotyping needs to account for sensor non-collection through design-based mitigation such as personal outreach by research staff or incentives for participation, a better qualitative understanding of the reasons for sensor non-collection at the individual-level, the continued development of additional statistical methods, and more reliance on within-subject over time study designs and data analyses. Within-subject analyses and study designs could leverage the observed high adherence, slow increase in sensor non-collection, and observed feasibility of long data collection periods.

Some statistical methods have been developed to mitigate the impact of missing data in digital phenotyping studies, especially for location data. For example, Barnett and Onnela^31^, proposed a weighted resampling approach, which resulted in a tenfold reduction in error, when compared to traditional linear interpolation, across several traditional mobility metrics. More recently, Liu and Onnela^32^ introduced a method for imputing GPS location traces that uses sparse online Gaussian Process, allowing for continuous, near real time imputation of missing data. To our knowledge, no imputation methods exist for raw accelerometer data.

Similarly, researchers should account for the level of sensor non-collection when performing power calculations and recruiting participants by either recruiting a greater number of participants to offset potential missing data or by leveraging within-subject designs and planning for a longer period of follow-up^34^. The ideal study length of follow-up will be determined by the phenomenon under investigation. While some research questions may benefit from many participants followed up for brief periods, other research questions may necessitate high-density, continuous GPS or accelerometer data collected over long periods of time. In the second case, it is often more statistically efficient to utilize a within-subject design with longer follow-up than a wide range of participants with limited follow-up. For example, using power calculations designed specifically for digital phenotyping based on Beiwe data^34^, we found that a smaller cohort of 50 participants followed over 180 days results in more statistical power than a larger cohort of 300 participants followed over 30 days (0.80 vs 0.74) despite the same number of person-days of observation (Supplementary Information Text S3).

Second, we found substantial individual-level variability in sensor non-collection relative to the study-level. This finding suggests that the observed large differences in sensor non-collection are not due to systematic study-related issues (e.g., data collection settings, issues with installing the app on participant phones, training of research staff on assisting participants with app and privacy settings) but are rather due to high between-person variability. Consistent with this finding, in alternative model specifications, we found more parsimonious models without study-level random or fixed effects to be nearly identical to the results presented here. The missingness appears to be independent of our measured, common demographic characteristics, and despite previously documented differences in smartphone usage (e.g., types of apps used by age or gender)^30^, it appears these differences in usage do not result in differential data collection in our sample. We note that, as with any study, there may be unobserved individual characteristics associated with missingness and thus detailed measurement of individual demographic factors is necessary to evaluate how missingness may affect specific outcomes of interest. Unmeasured, but likely important, individual-level factors include age or lifetime use of the phone and battery, charging habits, leisure activities such as hiking, camping, or other activities with where phone use is diminished. Such factors warrant future research.

Our study has several limitations. First, despite a large number of raw data measurements, measurement groupings, and person-days of observation, our sample still consisted of only six studies and 211 participants from 2015 to 2018. We estimated few statistically significant associations between missingness and demographic characteristics, but this finding could potentially be explained by lack of statistical power. This is the largest meta-study of digital phenotyping data collection; however, as digital phenotyping studies move beyond the pilot stage, similar meta-study approaches to understanding missingness across important sociodemographic covariates will continue be necessary. Similarly, the heterogeneity of participants across studies and homogeneity within studies may drive some of our findings. For example, 12 of the 33 black participants come from a single study of nurses, all of whom self-identified as female and skewed the distribution of gender in our sample. Thus, it is possible that our observed increased missingness among black participants is driven, at least in part, by occupation-related phone behaviors rather than by race/ethnicity. Differences between Android and iOS may be due to differences in the underlying userbase rather than software differences. In particular it appears that there may be a large socioeconomic difference between users of iOS and Android devices. Non-scientific market surveys have consistently found higher self-reported income among iOS users compared to Android users^35^, with one recent study reporting annual average salaries of approximately $53,000 and $37,000 for these two groups, respectively^36^. Previous market research suggests Black Americans are more likely to own Android devices than their White counterparts^37^. Fisher’s exact tests found no statistically significant differences between Android and iOS users across race/ethnicity or education in our data. Additional models, stratified by device type, show qualitatively similar results but large confidence intervals render interpretation of fixed effects inconclusive or difficult to interpret (Figure S2). Some subgroups may be more likely to own the latest phone and therefore own phones with greater battery capacity. Our models assume the number of missing measurement groupings follows a negative binomial distribution; however, the observed number of missing measurement groupings has an upper limit that may not follow the negative binomial distribution. To test the robustness of our results to this “ceiling effect,” we refit the primary model using a Bayesian hierarchical categorical regression, which makes no distributional assumptions on the number of missing measurement groupings, and found our results are robust to the type of model (Supplementary Information Text S4).

Despite these limitations, we believe our study is informative for future digital phenotyping studies. In summary, we believe our results indicate digital phenotyping is feasible across a large and diverse sample when coupled with careful study design and statistical analysis.

## Methods

### Data collection

This meta-study used data from six, independent studies. Five of the six studies were conducted in the state of Massachusetts with four studies comprised of undergraduate students at Harvard College (Studies A, C, E, and F); one study involved patients known to be at risk for mania and psychosis from McLean Hospital (Study B); and one study (Study D) consisted of an all-female subset of medical professionals in the Nurses’ Health Study 3^32^ with no diagnosed medical conditions. Study D is based in Massachusetts, but participants resided in several U.S. states. Each study received institutional review board (IRB) approval from their respective institutions for data collection (Table S2); another IRB approved by Harvard University governed the secondary analysis of the collected Beiwe data. Common inclusion criteria across all studies were: (1) ability to understand the English written consent form, (2) provision of written informed consent, (3) age 18 years or older, (4) possession of an Android or iOS smartphone, and (5) willingness to install the Beiwe application on their primary personal phone. Additional study-specific inclusion/exclusion criterion are listed in Table S2. In addition to obtaining informed consent from all participants, all methods were performed in accordance with relevant guidelines and regulations.

### Defining measurement groupings

The Beiwe Research Platform allows researchers to specify a sampling schedule separately for each sensor by adjusting the duration of the corresponding on-cycle and off-cycle. Using this information, we calculate the expected number of times the application attempts to collect data and the expected duration of data collection each day. However, ultimately the phone operating system controls data collection during an on-cycle and considers factors such as battery life and computational load when making this determination. To account for these design considerations, we aggregated the raw measurements into “measurement groupings,” which we defined as chunks of data that were collected within a researcher-specified on-cycle and were separated from the next measurement grouping by at least half of the researcher-specified off-cycle (Table S2; Fig. 2). Conceptually, a measurement grouping is an attempt by the smartphone application to collect data over some time interval, and it may have no observations (e.g., GPS was disabled by the participant) to several thousand (e.g., accelerometer data collection during a period of physical activity, such as running). Therefore, a missing measurement grouping (i.e., one with no observations), or sensor non-collection, could be due to (1) power management (e.g., low battery, a higher priority application is running, or high computational load); (2) sensor was disabled (e.g., activating airplane mode or deactivating GPS); or (3) the phone is off.

### Analysis

We used Bayesian hierarchical negative binomial regression to estimate the rate of sensor non-collection for GPS and accelerometer data. Unlike Poisson regression, negative binomial models allow for modeling both the mean and variance separately (i.e., allowing overdispersion), while the hierarchical framework accounts for the nested structure of the data (i.e., observations are clustered within users who are clustered within studies over time). For each user $i$ in study $j$, the distribution of the rate of sensor non-collection per day ${y}_{ij}$ is assumed to follow a negative binomial distribution. The mean of this distribution ${\mu }_{ij}$ is estimated as a log-linear function of $p$ individual-level covariates ${X}_{1ij}\dots {X}_{pij}$ with a study-specific offset ${E}_{j}$, the expected number of measurement groupings per day (a known, fixed value that results from the specification of on-cycle and off-cycle for each sensor). Further, due to the non-independence of daily observations within each user, we allow for a user-specific random intercept ${\gamma }_{0ij}$. Lastly, to account for potential clustering within studies, we allow for a study-specific random intercept ${\delta }_{0j}$. The model can be written as

$${y}_{ij} \sim \mathrm{NegBin}({\mu }_{ij}, \omega )$$

$$\mathrm{log}\left({\mu }_{ij}\right)= {\mathrm{log}(E}_{j})+{\alpha }_{0}+{\beta }_{1}{X}_{1ij}+\dots +{\beta }_{p}{X}_{pij}+{\gamma }_{0ij}+{\delta }_{0j}$$

$${\gamma }_{0ij}\sim \mathrm{Normal}\left(0, {\upsigma }_{\upgamma }^{2}\right)$$

$${\delta }_{0j}\sim \mathrm{Normal}\left(0, {\upsigma }_{\updelta }^{2}\right),$$

where the negative binomial distribution is parametrized in terms of the mean ${\mu }_{ij}$ and inverse overdispersion parameter ω^38^. Here ${\alpha }_{0}$ is the grand mean across all individuals, ${\delta }_{0j}$ is the study-specific deviation from the grand mean, and ${\gamma }_{0ij}$ is the individual-specific deviation from the study-specific mean. Both the study-level and individual-level random effects are assumed to be normally distributed with zero means. The variance parameters of the random effects, ${\upsigma }_{\upgamma }^{2}$ and ${\upsigma }_{\updelta }^{2}$, summarizes the variation in the rate of sensor non-collection at the individual- and study-level, after accounting for covariates. In addition, we estimated the fixed effects ${\beta }_{p}$ using covariates ${X}_{pij}$ at the individual level: duration in the study (in days), an indicator variable for operating system (iOS vs. Android), self-identified gender (male or female), educational attainment (less than four-year college degree or four-year degree and higher), race/ethnicity (non-Hispanic White, non-Hispanic Black, Asian, other race/multiple race/Hispanic, or American Indian / Alaskan Native), and age. Sensitivity analyses presented in Supplementary Information Text S2 indicate our results are robust to several alternative model specifications.

Models were fit using the No-U-Turn Sampler^39^, an efficient, adaptive Hamiltonian Monte Carlo algorithm. Specifically, we used the brm() function from the brms package^40^ which interfaces with the Stan library^41^. All parameters were assigned the default brms priors. Specifically, fixed effects were assigned an uninformative, improper prior $\beta \sim \mathrm{Uniform}\left(-\infty ,+\infty \right)$; the intercept was assigned the diffuse prior $\alpha \sim {\mathrm{Student}}^{\mathrm{^{\prime}}}\mathrm{s }t\left(3, 6.7, 2.5\right)$; and the standard deviation of the random effects were assigned the diffuse prior ${\upsigma }_{\upgamma }\sim \mathrm{Half}-{\mathrm{Student}}^{\mathrm{^{\prime}}}\mathrm{s }t\left(3, 0, 2.5\right)$. All models were fit using eight independent chains. Model convergence was assessed using the rank-normalized-split-$\widehat{R}$ and rank-normalized-folded-split-$\widehat{R}$, and the model was considered successfully converged when the maximum of both $\widehat{R}\le 1.01$. To ensure reliable posterior estimates, each chain was run until the Bulk Effective Sample Size and Tail Effective Sample Size metrics reached at least 100 samples per chain (Supplementary Information)^42^. We used the widely applicable information criterion (WAIC)^43^, the asymptotically-equivalent leave-one-out cross-validation^44^ with Pareto smoothed importance sampling (LOO)^45^, and Bayesian R-squared (Bayes ${R}^{2}$)^46^ to evaluate model goodness-of-fit, the necessity of random effects components, other nesting structures (e.g., observations within users or observations within studies), and other model specifications (Supplementary Information Text S2). All analyses were performed in R 4.0.2^47^.

## Supplementary Information

Supplementary Information.

## Data Availability

While this research use only metadata (e.g., timestamps of GPS pings rather than coordinates), dates of participant activity can be considered personally identifiable information; therefore, the data cannot be shared publicly. Deidentified, metadata used in this meta-study is available upon request, contingent upon appropriate IRB approvals or exemptions from participating institutions. While not the raw data, these data will provide sufficient information to reproduce our results (e.g., using shifted and/or adding noise to timestamps, re-randomized user identifiers). Replication code can be found at https://github.com/mkiang/beiwe_missing_data or https://github.com/onnela-lab/beiwe_missing_data (Supplementary Information Text S5). The Beiwe platform is open source and publicly available (Supplementary Information Text S1).
