# Supplementary material for: Sociodemographic characteristics of missing data in digital phenotyping
Source: Sci Rep. 2021 Jul 29;11:15408. doi: 10.1038/s41598-021-94516-7 (PMC8322366; doi:10.1038/s41598-021-94516-7)
Supplement: Supplementary file 1 — Supplementary Information. [file 41598_2021_94516_MOESM1_ESM.docx]

Supplementary Information: *Sociodemographic Characteristics of Missing Data in Digital Phenotyping*

Mathew V Kiang ScD, Jarvis T Chen ScD, Nancy Krieger PhD, Caroline O Buckee PhD, Monica J Alexander PhD, Justin T Baker MD PhD, Randy L Buckner PhD, Garth Coombs III PhD, Janet W Rich-Edwards ScD, Kenzie W Carlson BA, and Jukka-Pekka Onnela DSc^*^

*Corresponding Author:

Jukka-Pekka Onnela

*e-mail*: onnela@hsph.harvard.edu

*phone*: 617-432-1965

# Supplementary Tables

## Table S1. Summary statistics of sensor data by study.

Data are from six studies with a wide range in number of participants, time in study (person-days), number of raw data points collected (Obs.), number of measurement groupings (M.G.s), and percent of sensor non-collection (N.C.). Note that both Obs. and M.G.s are expressed in millions (M). For Studies A, B, C, E and F, the accelerometer on/off cycles were set for 10 seconds on / 10 seconds off. For Study D, the accelerometer on/off cycle was set for 10 seconds on / 1200 seconds off. For GPS, the on/off cycles, in seconds, were: Study A and C (90/1200); Study B and F (120/600); Study D (60/1200); and Study E (60/300).

|  | **Accelerometer** | | | | **GPS** | | | |
| --- | --- | --- | --- | --- | --- | --- | --- | --- |
|  | *Person-days (#)* | *Obs.* | *M.G.* | *N.C. (%)* | *Person-days (#)* | *Obs.* | *M.G.* | *N.C. (%)* |
| Study A | 2200.56 | 979.89M | 8.87M | 24.11 | 2122.81 | 10.93M | 0.17M | 33.73 |
| Study B | 4753.72 | 1186.09M | 12.91M | 38.24 | 4760.00 | 37.75M | 0.44M | 33.36 |
| Study C | 551.89 | 177.03M | 1.42M | 41.54 | 551.94 | 4.60M | 0.10M | 28.35 |
| Study D | 1893.02 | 13.96M | 0.10M | 28.78 | 1878.74 | 5.10M | 0.11M | 27.60 |
| Study E | 4667.43 | 1583.03M | 14.13M | 30.76 | 4643.05 | 14.89M | 0.32M | 22.54 |
| Study F | 15442.52 | 4206.92M | 41.55M | 38.11 | 15390.42 | 39.51M | 0.97M | 28.27 |
| **Total** | **29509.14** | **8146.92M** | **78.98M** | **35.37** | **29346.95** | **112.77M** | **2.12M** | **28.53** |

## Table S2. Institutional review board approval and inclusion/exclusion criteria.

Institutional review board (IRB) approval was granted for data collection for each study, by their respective institutions, with the following inclusion/exclusion criteria. In addition, for all studies, IRB approval was granted for the secondary data analysis to be performed at Harvard TH Chan School of Public Health (IRB16-0966).

| **Study** | **IRB Protocol Number (Institution)** | **Additional Inclusion/Exclusion Criteria** |
| --- | --- | --- |
| A and F | IRB16-1230 (Harvard) | Inclusion: Normal, cognitively intact college students; 18-28 years old; native of fluent English speakers; recruitment focused on freshmen |
| C | 2015P002189 (Massachusetts General Hospital) | Inclusion: Must have severe affective and psychotic illness; over the age of 18 |
| D | IRB16-0440 (Harvard) | Inclusion: Normal, cognitively intact college students; 18-22 years old; native of fluent English speakers; students enrolled in exam-based science courses (any grade)  Exclusion: Contraindications for MRI |
| E | 2006P000473 (Brigham and Women’s Hospital) | Inclusion: Must be a current participant of the Nurses’ Health Study 3 |
| G | IRB15-3613 (Harvard) | Inclusion: Normal, cognitively intact college students; 18-22 years old; native of fluent English speakers; students enrolled in exam-based science or math courses (any grade) |

# Supplementary Figures

## Figure S1. Periods of data collection for each study and each participant.

Each horizontal line represents a single study participant with the endpoints at the first and last day of observation, and the intensity of color represents the amount of data collection per day such that darker tones have higher sensor non-collection (i.e., more missing data). Studies varied in number of participants, length of observation, and rate of attrition. Each study is represented by a different color. Note that because dates of study participation may be considered personally identifiable information, time (x-axis) is represented as days relative to the earliest date and not calendar time. All studies occurred between 2015 and 2018.

## Figure S2. Fixed effects estimates from models stratified by device operating system.

Here, we present the results from our final model stratified for devices that use iOS (orange) or Android (green) with the primary model (including both operating systems) as a reference (purple). Large confidence intervals in the Android only model prevent us from drawing any strong conclusions about potentially discordant results.


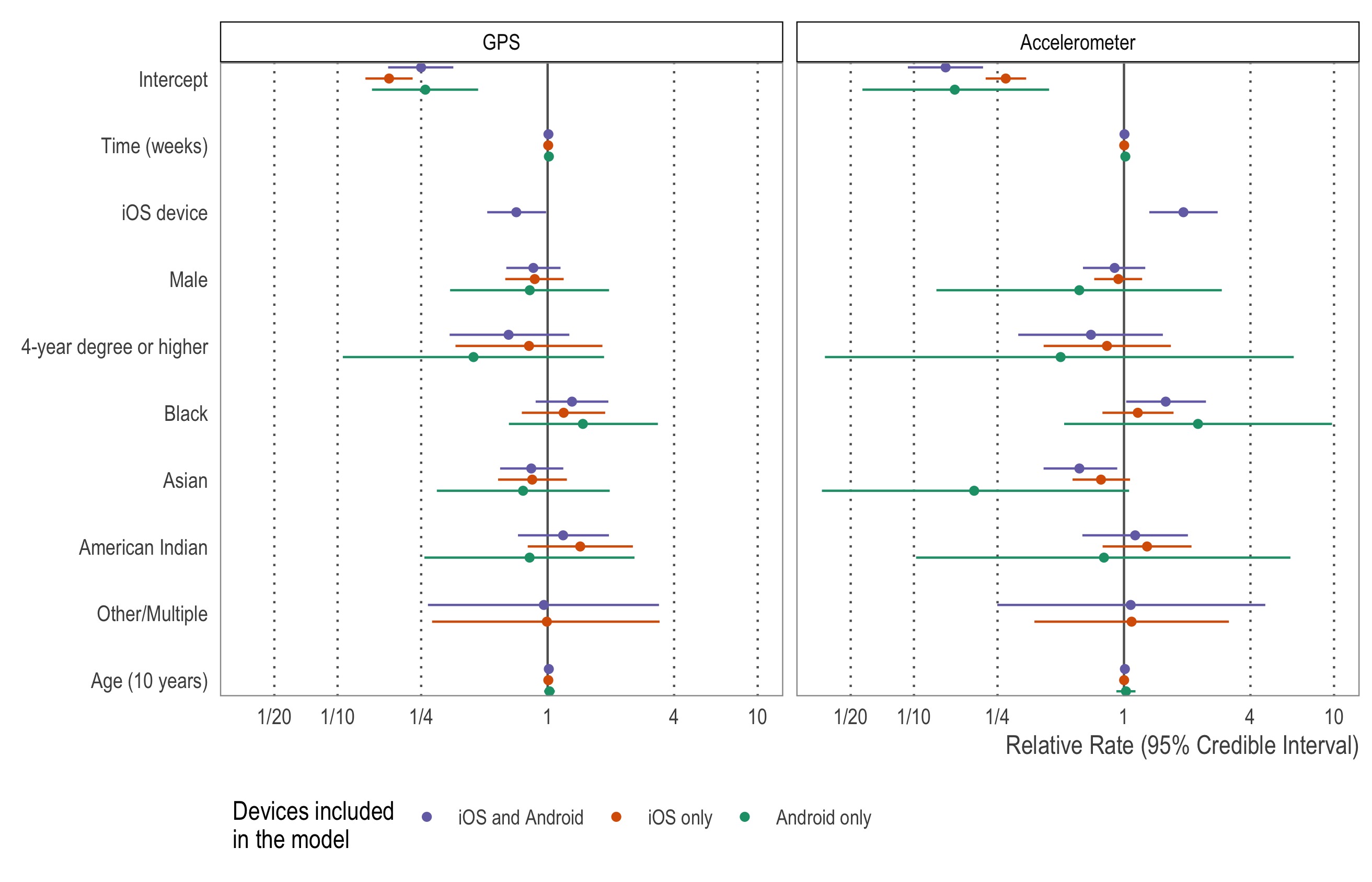


# Supplementary Text

## Text S1. Open-source code repositories for Beiwe and information about the platform.

There are three affiliated code repositories for the Beiwe Research Platform. The Beiwe Research Platform is powered by backend code that can be found at <https://github.com/onnela-lab/beiwe-backend>. The Beiwe Android frontend code can be found at <https://github.com/onnela-lab/beiwe-android>. The Beiwe iOS frontend code can be found at https://github.com/onnela-lab/beiwe-ios. More information about Beiwe can be found at <https://www.beiwe.org>.

## Text S2. Assessing goodness-of-fit with alternative model specifications.

Given the structure of the data, several alternative models (with different assumptions) can be reasonably specified: (1) a non-hierarchical model, (2) a non-hierarchical model with study fixed effects, (3) a two-level hierarchical model with observations nested in users (but not in studies), (4) a two-level hierarchical model with observations nested in users and study fixed effects, and (5) a two-level hierarchical model with observations nested in study. Here, we compare these alternative model specifications to the model presented in the paper (two-level model with observations nested in users).

Consistent with the model in the main text (three-level hierarchical model with observations nested in users nested in studies), assume for each user $i$ in study $j$, the rate of sensor non-collection per day $y_{ij}$ follows a negative binomial distribution. The mean rate of sensor non-collection $\mu_{ij}$ is estimated as a log-linear function of $p$ covariates $X_{1ij}\ldots X_{pij}$ with a study-specific offset $E_{j}$, the expected number of measurement groupings per day (a known, fixed, value). Further, due to the non-independence of daily observations within each user and clustering of users within studies, we allow for a user-specific random intercept $\gamma_{0ij}$ and a study-specific random intercept $\delta_{0j}$. This model can be written as

$$y_{ij} \sim\mathrm{NegBin}(\mu_{ij}, \omega)$$

$$\log\left( \mu_{ij} \right)= {\log(E}_{j})+\alpha_{0}+\beta_{1}X_{1ij}+\ldots+\beta_{p}X_{pij}+\gamma_{0ij}+\delta_{0j}$$

$$\gamma_{0ij}\sim\mathrm{Normal}\left( 0, \sigma_{\gamma}^{2} \right)$$

$\delta_{0j}\sim\mathrm{Normal}\left( 0, \sigma_{\delta}^{2} \right)$,

where the negative binomial distribution is parametrized in terms of the mean $\mu_{ij}$ and inverse overdispersion parameter ω. A reasonable, simpler specification would be to set both $\sigma_{\delta}^{2}$ and $\sigma_{\gamma}^{2}$ equal to zero for a non-hierarchical model. This non-hierarchical specification could also include study fixed effects in addition to the covariates outlined in the manuscript. We could specify a two-level model with observations nested in users and add study fixed effects (i.e., as covariates) or the same model without study fixed effects. Alternatively, we could specify a two-level model but set $\sigma_{\gamma}^{2}=0$ so observations are nested in studies (but not users). Recall that the primary model presented in the text is the above model with all parameters to be estimated. A summary of these models is shown below with their goodness-of-fit metrics and the primary model in bold.

|  | User random effect | Study random effects | Study fixed effects | **Accelerometer** | | **GPS** | |
| --- | --- | --- | --- | --- | --- | --- | --- |
|  |  |  |  | Δ LOO (SE Δ) | Δ WAIC (SE Δ) | Δ LOO (SE Δ) | Δ WAIC (SE Δ) |
| Non-hierarchical model |  |  |  | -3,476.4 (90.7) | -3,478.4 (90.4) | -3,828.3 (95.3) | -3,829.7 (95.2) |
| Non-hierarchical model with study fixed effects |  |  | X | -3,280.4 (88.4) | -3,281.9 (88.1) | -3,725.7 (94.5) | -3,738.0 (94.0) |
| Two-level model with user random effects | X |  |  | -0.3 (0.7) | -0.0 (0.0) | -0.2 (0.5) | -0.2 (0.5) |
| Two-level model with user random effects and study fixed effects | X |  | X | 0.0 (0.0) | -0.7 (0.8) | 0.0 (0.0) | 0.0 (0.0) |
| Two-level model with study random effects |  | X |  | -3,280.5 (88.4) | -3,282.0 (88.1) | -3,736.7 (94.5) | -3,737.8 (94.4) |
| **Three-level model with user and study random effects** | **X** | **X** |  | **-0.3 (0.4)** | **-0.4 (0.6)** | **-0.5 (0.3)** | **-0.5 (0.3)** |

For each model, we calculated the goodness-of-fit using Pareto smoothed leave-one-out cross-validation (LOO) and the asymptotically-equivalent widely applicable information criterion (WAIC). For each sensor, we calculated the difference in the theoretical expected log pointwise predictive density of each model relative to the best performing model (ΔLOO and ΔWAIC) and the standard error of this quantity (SE Δ). As shown above, the three-level model with user and study random effects, the two-level model with user random effects and study fixed effects, and the primary model (two-level with user random effects) performed substantially better than the alternative parameterizations. For both sensors, the primary model used in the manuscript was either the best performing model or was within 2 standard errors of the best performing model. We selected the three-level model because it has the most theoretical backing (i.e., variation comes at both the user and the study level), but we note that parameter estimates do not change when using the other two specifications and more parsimonious models exist. See the online supplement for all model estimates (Text S5).

## Text S3. Sample size calculations.

To assess the impact of sample size vs duration of following, we used the online supplemental application, “Digital Phenotyping Power Calculation”, available at <https://onnela-lab.shinyapps.io/digital_phenotyping_sample_size_calculator/>. This application is based on the following paper: Barnett, I., Torous, J., Reeder, H. T., Baker, J., & Onnela, J. P. (2020). Determining sample size and length of follow-up for smartphone-based digital phenotyping studies. Journal of the American Medical Informatics Association, 27(12), 1844-1849.

We assumed 75% of data were missing and that the variance of the random effect was 0.8 with a Type 1 error rate of 0.05 and daily observations. Under these conditions, a cohort of 50 participants followed over 180 days (9,000 person-days of observation) would result in statistical power of 0.80 and a cohort of 300 people followed for 30 days (9,000 person-days of observation), would result in power of 0.74. Changing either the rate of missingness or the variance of the random effect does not change the qualitative result that, for a fixed number of person-days of follow-up, fewer participants over longer periods of time generally have higher statistical power compared to more participants over shorter periods of follow-up.

## Text S4. Testing the impact of the “ceiling effect” on our models.

The models presented in the main text and the Supplemental Text S2 are based on the negative binomial distribution. However, our data have an upper limit (or ceiling) because missing measurement groupings per day can only be as high as the expected number of measurement groupings per day. To assess the sensitivity of our results to this “ceiling effect”, we refit our primary model using a Bayesian hierarchical categorical regression with observations nested in users nested in studies. The beta coefficients are presented below with low levels of missingness (0 to 10%) as the reference. The full numerical results are available in the online repository (Text S5). We note that our results are robust to the model type.

##
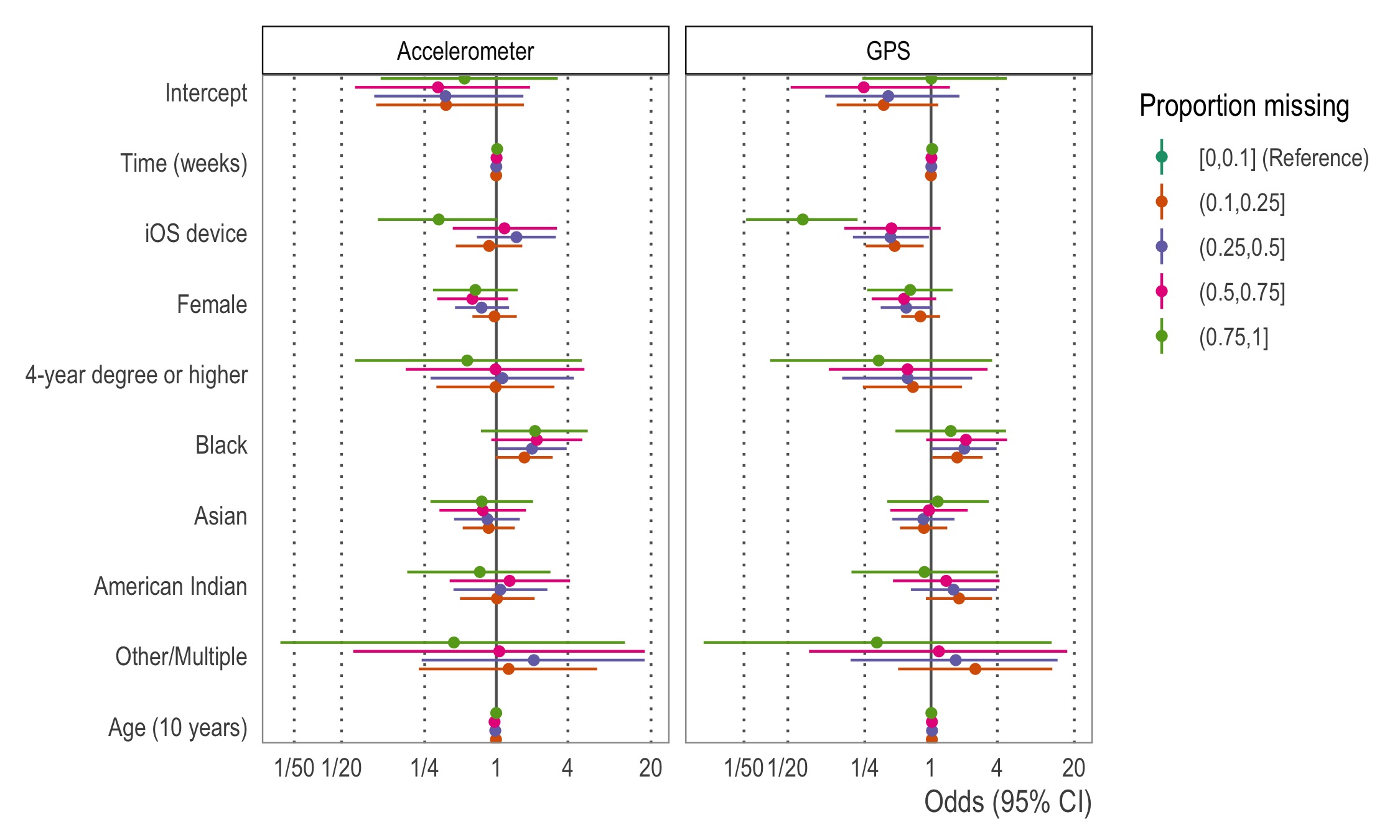
Text S5. Data availability and replication code.

While this research use only metadata (e.g., timestamps of GPS pings rather than coordinates), dates of participant activity can be considered personally identifiable information; therefore, the data cannot be shared publicly. Deidentified, metadata used in this meta-study is available upon request, contingent upon appropriate IRB approvals or exemptions from participating institutions. While not the raw data, these data will provide sufficient information to reproduce our results (e.g., using shifted and/or adding noise to timestamps, re-randomized user identifiers). Replication code can be found at https://github.com/mkiang/beiwe_missing_data or https://github.com/onnela-lab/beiwe_missing_data (Supplementary Information Text S5). The Beiwe platform is open source and publicly available (Supplementary Information Text S1).

The code and documentation are near exact copies of the code used in this project with only minor differences. Specifically, for this paper, we use internal study project names which may include a year and/or month. Out of an abundance of caution, we remove any references to these study names. However, the code is otherwise the same. Full model results are also provided in the online repository.

Please see the repository at: https://github.com/mkiang/beiwe_missing_data or https://github.com/onnela-lab/beiwe_missing_data.
